# Endometriosis and Adolescence: The Impact of Dysmenorrhea

**DOI:** 10.3390/jcm12175624

**Published:** 2023-08-29

**Authors:** Francesco G. Martire, Emilio Piccione, Caterina Exacoustos, Errico Zupi

**Affiliations:** 1 Department of Biomedicine and Prevention, Section of Gynecology and Obstetrics, University of Rome “Tor Vergata”, 00133 Rome, Italy; francescogmartire@libero.it; 2 Department of Molecular and Developmental Medicine, Obstetrics and Gynecological Clinic, University of Siena, 53100 Siena, Italy; ezupi@libero.it; 3 Department of Surgical Sciences, Catholic University “Our Lady of Good Counsel”, 1000 Tirane, Albania; 4 Department of Surgical Sciences, Section of Gynecology and Obstetrics, University of Rome “Tor Vergata”, 00133 Rome, Italy; caterinaexacoustos@tiscali.it

**Keywords:** adolescence, diagnosis, dysmenorrhea, endometriosis, management

## Abstract

Endometriosis affects approximately 10% of premenopausal women worldwide. Despite its impact on quality of life, the delay in diagnosing this chronic disease is well known. Many patients with endometriosis report having suffered from dysmenorrhea and chronic pelvic pain in adolescence or at a young age. However, this painful symptom is often highly underestimated and considered a normal and transient symptom in young women. The real prevalence of endometriosis in adolescence remains uncertain. Some authors recently described at least one ultrasound feature of endometriosis in 13.3% of a general population of adolescent girls, which increased to 35.3% in young girls with severe dysmenorrhea. Dysmenorrhea is classified as primary dysmenorrhea or secondary dysmenorrhea. Primary dysmenorrhea is defined as a menstrual pain without organic disease, while secondary dysmenorrhea is defined as a menstrual pain associated with organic pelvic pathology. Since endometriosis represents the main cause of secondary dysmenorrhea in adolescents and young women, it is important to determine whether the patient has primary dysmenorrhea or additional suggestive symptoms related to endometriosis. Endometriosis in adolescent patients is a challenging problem with clinical and pathological differences compared with its presentation in premenopausal women. Adolescents and young women with dysmenorrhea and painful symptoms that suggest endometriosis should be referred to dedicated endometriosis centers for an early diagnosis and appropriate medical and surgical management. This paper aims to describe the role of dysmenorrhea in adolescents and the management of these young patients to confirm or exclude endometriosis.

## 1. Introduction

Endometriosis affects around 10% of premenopausal women worldwide. However, the true prevalence of endometriosis in adolescence remains uncertain.

Although the clinical signs of the disease, such as painful symptoms during menstruation, may appear early after menarche, frequently endometriosis is suspected late, resulting in a diagnostic delay, often years after onset [1].

Many patients with endometriosis report having suffered from dysmenorrhea when adolescents or at a young age [2]. Dysmenorrhea has a great impact on adolescents’ lives, causing restrictions of daily activities [3], reductions in academic performance [4], and reductions in the quality of sleep [5], as well as negatively affecting mood, causing anxiety and depression.

Dysmenorrhea is classified as primary dysmenorrhea or secondary dysmenorrhea.

Primary dysmenorrhea is defined as a pain during menstruation without organic disease, while secondary dysmenorrhea is defined as a pain during menstruation associated with organic pelvic pathology.

Since endometriosis represents the main cause for secondary dysmenorrhea in adolescents and young women, it is fundamental to understand whether the patient has primary dysmenorrhea or additional suggestive symptoms related to endometriosis [6].

Dysmenorrhea is often considered as an inevitable manifestation of menses. Therefore, in speaking with adolescents, their parents, and or their caregivers, this painful symptom is highly underestimated and evaluated as a normal condition [7].

Endometriosis in adolescents and young women is a difficult problem to interpret with clinical and pathological differences compared with premenopausal women.

Therefore, adolescents and young women with dysmenorrhea and painful symptoms that suggest endometriosis should be referred to dedicated endometriosis centers for an early diagnosis and appropriate medical and surgical management.

## 2. Primary Dysmenorrhea

Primary dysmenorrhea the most common menstrual symptom in adolescents and young women [8,9]. It is defined by presence of recurrent lower abdominal pain during menses, and is the main cause a girl decides to book a gynecologic visit. Adolescents usually describe this type of pain as mild or moderate [10].

Despite the reduction in the quality of life due to this symptom, the prevalence of primary dysmenorrhea is underestimated.

According to the literature, the prevalence of the primary dysmenorrhea in adolescents ranges between 45% and 95%. However, the prevalence is underestimated because women often consider pain to be a normal part of menstruation, and therefore, do not refer it, and do not feel the need to consult a doctor to investigate the condition and to seek medical care [11]. Primary dysmenorrhea occurs generally within 6–12 months from menarche. It is usually present during the first or second day of menses and begins just before the start of menstruation.

Pain is associated with increased release of prostaglandins at the time of menstruation; in fact, higher prostaglandin levels were found in endometrial tissue and menstrual fluid of women with dysmenorrhea compared with asymptomatic women [12].

This increase in the levels of prostaglandins during menstruation could lead to both myometrial hypercontractility, resulting in hypoxia and ischemia of uterine muscle and the perception of pain, and systemic symptoms often associated with dysmenorrhea, such as nausea, diarrhea, and fatigue.

Pre-sexually active adolescents and young women who report primary dysmenorrhea without other suggestive symptoms related to endometriosis should receive empirical medical treatment to reduce and or eliminate the painful symptoms. However, if the painful symptomatology persists despite treatment, further evaluation to exclude secondary causes is necessary, including pelvic examination and imaging.

## 3. Secondary Dysmenorrhea

Secondary dysmenorrhea is characterized by menstrual pain in the presence of organic disease It can arise from gynecological or non-gynecological causes. In the former case, the main pathologies associated with the occurrence of secondary dysmenorrhea are endometriosis, myometrial pathologies such as adenomyosis and fibroids, postoperative adhesion syndrome, pelvic inflammatory disease (PID), ovarian cyst, hematocolpus due to obstructive mullerian abnormalities, and hydrosalpinx; while in the second case, when the origin is not gynecological, the pain often results from gastrointestinal diseases or urinary tract diseases.

For a complete evaluation of these types of conditions, a multidisciplinary approach should be considered.

To confirm the presence of organic disease, it is important to perform an accurate anamnesis evaluating every aspect of menstruation, such as time of menarche, duration of bleeding, intervals between menses, amount of menstrual flow by pictorial blood loss assessment chart (PBAC), and related symptoms. The main symptoms to be evaluated are pain, nausea, diarrhea, and fatigue; it is also important to carefully assess when the symptoms arise, the pain intensity, and the impact on everyday activities. In addition, an extensive laboratory evaluation, including complete blood count, C-reactive protein, metabolic panel, and urinalysis with urine culture may be helpful. If associated bowel symptoms are also found, the physician should also consider various organic gastrointestinal diseases.

It is important to evaluate growth delay, weight loss, bloody diarrhea, fecal calprotectin, circulating antibodies against tissue transglutaminase, and HLA typing. Finally, a complete endoscopic evaluation could be useful to perform a differential diagnosis between these pathologies.

## 4. Dysmenorrhea and Endometriosis

Endometriosis represents the main cause of secondary dysmenorrhea among adolescent and young women. Often, it is accompanied by other symptoms related to endometriosis, such as dyspareunia, chronic pelvic pain (CPP), dyschezia, dysuria, and heavy menstrual bleeding.

The onset of secondary dysmenorrhea usually begins a few years after menarche and is more common after 20 years of age.

According to the ESHRE guidelines [13], indicative manifestations of endometriosis include the primary symptoms, as well as acyclic pain, bowel functional symptoms, genitourinary symptoms, and resistance to empirical medical treatment (NSAIDs and hormonal therapy).

Some authors recently observed the prevalence of ultrasound (US) features of endometriosis in 13.3% of a population of adolescents (12–20 years) who underwent ultrasound evaluation by the endocavitary approach (transvaginal sonography or transrectal sonography) for several conditions [14]. The authors also described dysmenorrhea as a risk factor for endometriosis; in fact, in the presence of this symptom, the percentage of US signs of endometriosis increased up to 21%. At the same time, other authors observed that the prevalence of endometriosis in young women with dysmenorrhea and chronic pelvic pain (CPP) ranged between 25% and 73% [15,16]. An important role could be played by severe dysmenorrhea (VAS score ≥ 7), which in fact could be suggestive of secondary dysmenorrhea, particularly of endometriosis. According to these data, among young women with severe dysmenorrhea, a prevalence of US signs of endometriosis of 35.3% detected by TVS or TRS scan [17]. Furthermore, patients with US findings of endometriosis have other associated symptoms, such as dyspareunia, gastrointestinal symptoms, and HMB [17]. In fact, the patients with endometriosis report more painful symptoms associated with dysmenorrhea than those without endometriosis; therefore, these painful symptoms reported by patients increase the likelihood of the presence of endometriosis disease.

With respect to the literature, the presence of the above outlined painful symptoms in adolescents and young women associated with school or work absenteeism should not be underestimated and could be considered disease markers [18,19]. In the presence of these indicative conditions, the clinician should perform a careful physical pelvic examination and TVS or TRS evaluation to detect any sign of disease, both clinical and ultrasound, which can often be minimal and isolated.

## 5. Dysmenorrhea and Adenomyosis

Adenomyosis is a chronic disease, defined by the presence of ectopic endometrial and stromal tissue within the myometrium, that affects the uterus and is characterized by various symptoms, including dysmenorrhea, heavy menstrual bleeding (HMB), dyspareunia, pelvic pain, and infertility [20].

The prevalence of adenomyosis in premenopausal women is uncertain. We know that the prevalence based on hysterectomy samples is ranges between 15 and 60% [21].

However, we still have limited data concerning the presence of adenomyosis in adolescent patients [20]. Adenomyosis, such as endometriosis, can manifest itself from a young age [22] and can have repercussions for life quality [23,24].

In fact, adenomyosis is not only a disease of premenopausal women (adulthood) but also affects adolescents and young women, with potential clinical implications. The main difference in the disease in adolescents and adults is its degree and form, with mostly mild or moderate and focal forms in adolescents [25]. Adenomyosis may start in adolescence but show clinical manifestations only in adulthood, probably because the disease starts as a mild form.

The diagnosis is achievable by noninvasive methods such as ultrasound or MRI [25].

Ultrasonographic features of endometriosis and adenomyosis in adolescents are more frequent than we expect. In fact, according to a previous study [14] which observed a prevalence of 13.3% of endometriosis in adolescent patients, US signs of adenomyosis were detected in 5.2% of cases, and in 44% of cases, adenomyosis was associated with endometriosis. The symptoms related to endometriosis and adenomyosis are often similar and overlap; therefore, this symptomatology could also occur in presence of adenomyosis alone. As a result, performing a diagnostic laparoscopy in adolescents with dysmenorrhea and chronic pelvic pain not responsive to medical treatment could misdiagnose adenomyosis, especially in mild forms.

Therefore, an ultrasound scan seems to be the best noninvasive method to detect adenomyosis and reduce misdiagnose. However, it still requires expert sonographers to make an accurate diagnosis of focal and mild to moderate forms.

Given that pelvic endometriosis and adenomyosis often coexist, the diagnosis should be made by an abdominal and pelvic examination. The physical signs include an enlarged uterus and tenderness. For diagnosis using ultrasound (performed by the transvaginal method in sexually active patients or transrectally in pre-sexually active patients), the signs should include specific ultrasonographic features, such as an irregular or interrupted junctional zone, myometrial ‘cysts’, subendometrial lines or ‘buds’, presence of fan-shaped shadowing, uterine wall asymmetry, an increased myometrial vascularity, and an enlarged uterus. MRI can be used in cases where ultrasound is not possible or in cases of doubtful interpretation, but should always be secondary to ultrasound [13]. It is important to remember that fibroids and adenomyosis may show a similar clinical presentation as they are both estrogen-dependent diseases, and that hormone therapy makes ultrasound diagnosis more difficult, due to changes in US features [26].

## 6. Differential Diagnosis

Bowel involvement in patients with endometriosis ranges between 2 and 46% [27] and is less frequent in adolescents than in adults [28]. However, there are many intestinal diseases with painful symptoms similar to endometriosis, such as Irritable Bowel Syndrome (IBS), inflammatory bowel diseases (IBD), and celiac disease that can also be considered risk factors for endometriotic disease. Irritable Bowel Syndrome (IBS), a chronic disease involving the large bowel and characterized by visceral hypersensitivity, is five times more frequent than in the general female population [29]. For this reason, when adolescents present bowel symptoms, the clinician should consider the presence of various organic gastrointestinal diseases. The prevalence of Inflammatory Bowel Diseases (IBD) in children and adolescents is around 20% [30] and are frequently characterized by delays to puberty and growth, weight loss, and bloody diarrhea (more frequent in ulcerative colitis than in Crohn’s disease). In 25% of patients, the presence of extra-intestinal symptoms is possible. In the evaluation of IBD, fecal calprotectin can be considered a useful tool for screening [31], but all adolescents with clinical suspicion of IBD should receive an endoscopic assessment of the gastrointestinal tract, with multiple biopsies required for diagnosis. To recognize whether the small or proximal large intestine is involved in Crohn’s disease imaging may also be helpful [32]. The prevalence of celiac disease in adolescence ranges between 1 and 3% of the general Caucasian population [33], and over 40% of these patients have both classic gastroenterologic signs (chronic diarrhea and undigested food in the stool) and other features, such as hypothyroidism, diabetes, chronic anemia, or delayed menarche [34]. Most patients have circulating antibodies against tissue transglutaminase, and therefore, HLA typing is useful as a rule-out test [35]. For the diagnosis, it is necessary to show the presence of duodenal villous atrophy by endoscopy. In adolescents, according to European guidelines, the diagnosis may be reached without duodenal biopsy as long as rigorous symptomatic and serologic criteria are fulfilled [36]. Meckel’s diverticulum [37] or subacute/chronic appendicitis [38] are less common causes of intestinal symptoms in adolescents. Congenital uterine anomalies are another condition to evaluate in the context of making a differential diagnosis for endometriosis because the painful symptoms and abnormal bleeding at the time of menarche often overlap with the clinical manifestations of endometriosis. The outflow tract may be either obstructed or unobstructed by anatomical defects. Obstruction of the outflow tract causes pain, particularly amenorrhea and progressive pelvic pain, while the unobstructed outflow tract is usually pain free.

The incidence of reproductive tract anomalies is estimated to affect around 6% of women [39].

Therefore, in the presence of the symptoms and signs outlined above, it is necessary to perform a pelvic evaluation to confirm or exclude an outflow tract obstruction, although this may be challenging for a young girl. In contrast, pelvic examination in patients with nonobstructive abnormalities shows regular external genitalia and a normal hymen. The second step to evaluate reproductive tract anomalies is a pelvic ultrasound, while the gold standard for accurately delineating the presence, size, and anatomy of the uterus and other associated abnormalities is MRI [10,40].

## 7. Diagnosis

In all adolescents who present with dysmenorrhea, it is important to perform an accurate anamnesis, a physical examination, an ultrasonographic evaluation and/or magnetic resonance imaging.

During anamnesis, it is fundamental to determine the age of first menstruation, menstrual cycle characteristics, previous surgical treatment, autoimmune and endocrinological diseases, and familial history of endometriosis. Furthermore, the clinician should ask about any medication during the menstrual periods, such as non-steroidal anti-inflammatory drugs (NSAIDs) and estro-progestin therapy, to assess the symptom severity. With respect to the ESHRE guidelines [13], is important to investigate and not underestimate suggestive conditions related to endometriosis, including early menarche, familial history of endometriosis, painful symptoms resisting empirical medical treatment, heavy menstrual bleeding, gastrointestinal and genitourinary symptoms, as well as associated symptoms including nausea, fatigue, and effects on daily activities.

The second step includes performing a physical examination with vaginal and/or rectal examination. Prior to such investigations, the clinician should discuss with the adolescent and her caregiver if they are acceptable. Often adolescents and their parents accept these examinations according to age and cultural background. However, no evidence was found regarding clinical examination in adolescents [13], so the clinician should decide on a case-by-case basis. After the clinical and physical examination, the diagnostic process should continue with instrumental evaluation, particularly by ultrasound and magnetic resonance imaging (MRI). However, most of these adolescents and young women are pre-sexually active, which is a significant constraint to ultrasound evaluation. This aspect requires a transabdominal method to investigate the pelvis or a transrectal approach.

The accuracy of the transabdominal technique is limited. The effectiveness of this method to detect all endometriosis features and localizations is limited. It is useful to detect ovarian endometriotic lesions [41] but cannot detect non-ovarian disease such as deep infiltrating endometriosis features [42].

Therefore, to detect deep infiltrating pelvic endometriosis lesions or adhesions when there is clinical suspicion of posterior compartment involvement, a transvaginal method (or transrectal ultrasound approach in the case of non-sexually active patients) is the most effective imaging technique. Furthermore, the accuracy improves if performed by an expert operator, especially one specializing in the field endometriosis [43].

To analyze the prevalence of endometriosis features in the adolescent population and recognize all possible patterns and localizations of the disease, it is crucial to choose an appropriate instrumental approach, especially given the need to use noninvasive tools for diagnosis. According to the literature, transvaginal ultrasonography (TVS) is the first-line technique to detect all different features of endometriosis, such as endometriomas and DIE, including involvement of the utero-sacral ligaments (USL), rectovaginal septum (RVS), and torus [44]. However, the transvaginal approach can often be impossible in pre-sexually active adolescent girls or in the presence of vaginal pathology as hypoplasia or agenesis. In these cases, the transrectal sonography (TRS) should be regarded as an excellent alternative. MRI is recommended if either patients or their relatives object to transrectal evaluation.

Magnetic Resonance Imaging (MRI) represents a non-invasive technique with high diagnostic accuracy that can be very useful to detect all features and sites of disease, with the additional advantage of not being an operator-dependent technique.

However, MRI cannot be indicated as the first choice for investigation. In fact, it should be considered a second-tier examination for the evaluation of endometriosis, both in adolescents and adults, because it is more expensive than US evaluation and is a diagnostic investigation performed by radiologists. In the presence of expert operators, the diagnostic accuracy of the two techniques can be considered similar.

Transvaginal ultrasonography can be considered the main technique to reveal and assess ovarian endometriotic cysts, whereas MRI is more useful for differentiating between superficial ovarian implants and endometriotic cysts. Moreover, in the presence of atypical lesions, it can provide supplementary information to exclude malignancy [45]. Nevertheless, it may be beneficial to investigate deep infiltrating endometriosis (DIE) [46]; in fact, the MRI technique displays a high degree of accuracy in identifying anterior and posterior endometriosis, especially for anterior signs of DIE [47].

Serum biomarkers (e.g., CA-125) are not considered for diagnosis or ruling out endometriosis, either in adolescents or in premenopausal women. In fact, CA125 levels do not discriminate between endometriosis patients and non-endometriosis patients [48].

It is likely that the inflammatory and autoimmune nature of endometriosis may be useful in the future for identifying other markers, such as complement proteins, especially in early forms such as those of adolescents [49].

According to the ESHRE recommendations [13], performing surgery to confirm an endometriosis disease diagnosis is considered inappropriate, both for adults and adolescents.

## 8. Treatment

The aim of medical therapy in adolescents and young women with endometriosis is to improve the painful symptomatology, block or at least reduce the disease progression, and protect future fertility [10].

The ESRHE guidelines [13] advise clinicians to treat adolescent girls with hormonal contraceptives or progestins to reduce endometriosis-related pain. However, despite all the hormonal contraceptives available today, there is no consensus on the appropriate therapy to be administered by age and patient type with respect to the endometriosis features.

Among the drugs that can be used as different lines of treatment, there are progestins, estro-progestins in continuous or cyclical mode, and GnRH agonists.

Progestin therapy includes different molecules, and it is likely that Dienogest and Norethindrone acetate are effective and do not create tolerability problems in adolescents and young women. In the medical management of disease, Dienogest is effective in reducing painful symptomatology related to endometriosis, improving the quality of life of patients, and often reduces the size of endometriotic lesions and their vascularization. These effects occur through its progestogenic and antiestrogenic action on both eutopic and ectopic endometrium [50]. Regarding the antiestrogenic action of Dienogest, side effects in adolescents and young women have been observed, such as symptoms of estrogen deprivation, so in presence of these symptoms, it could be useful to switch to estro-progestin therapy.

The use of oral contraceptives (estro-progestin therapy) should have the same effectiveness as progestin therapy; however, in patients with endometriosis who report dysmenorrhea as the main symptom, the administration in continuous mode should result in greater symptomatic benefits than cyclic administration [13].

As an alternative to oral administration, a subcutaneous implant of etonogestrel (ENG) can be used with good results in terms of reducing painful symptoms, despite the limited experience in adolescents.

Finally, LNG-IUS could be a viable alternative in sexually active adolescents and young women in whom endometriosis and adenomyosis coexist.

The use of GnRH agonists should be considered as a second line of treatment. In fact, it is only permissible in adolescent girls with confirmed endometriosis (diagnosed laparoscopically) when the patient is refractory to further medical (hormonal contraceptives or progestin therapy) or surgical treatment. The mechanism underlying their function is hypothalamic–pituitary axis suppression, which leads to a hypoestrogenic milieu. To reduce collateral effects, add-back therapy and adequate monitoring of bone mineral density might be useful [51].

Certainly, the development of new molecules that could reduce the size of disease foci or block its progression is a fundamental objective. The use of new therapeutic agents, such as Ankaferd Blood Stopper (ABS) and oxytocin (OT) are showing great potential [52]. Finally, therapy must be individualized, and there is no single best treatment for endometriosis in adolescent girls.

Surgical treatment should be considered only for specific conditions. Particularly, in adolescents, we must never forget the importance of preserving fertility and hormonal function. Therefore, surgical treatment should be considered only in cases with rapid growth of an ovarian endometriotic cyst, in cases of a suspicious ovarian cyst in which the risk of malignancy cannot be excluded by ultrasound or MRI, in cases with persistence of pain symptoms despite hormonal treatment, or in the presence of urinary and bowel complications [13].

Surgery should be performed to eliminate visible signs of the disease and reconstitute the anatomy, improve the quality of life and facilitate a future spontaneous pregnancy [10].

Regarding the surgical management of the endometriomas, it is important when performing all surgical procedures, to reduce the damage to healthy tissue and to preserve the ovarian reserve.

The stripping technique is a valid surgical procedure that allows good surgical results with reduced recurrence rates. When the stripping technique is performed, it is very important to identify the correct cleavage plane to reduce the ovarian damage and preserve healthy ovarian tissue. During the procedure, hemostasis should be performed by accurate cauterization or by atraumatic sutures not involving the ovarian hilus. Another useful surgical procedure is CO2 laser vaporization of the cystic capsule. This surgical technique has the advantage of being safe and easily reproducible, and is especially effective at reducing ovarian damage [53].

It is important to know that in adolescents and young women, endometriotic implants are often present as clear lesions, observable as shiny peritoneal vesicles. During surgery is not easy to identify these small lesions, and this difficulty could result in diagnostic and treatment delay, with deterioration in the quality of life [15,54]. At the same time, radical superficial endometriosis excision could enhance the development of adhesions responsible for the persistence of painful symptoms. Considering these factors, radical excisional surgery should not be used in adolescents [55].

Pelvic pain in adolescents can be treated surgically as in the adult population [56], but it is still associated with a higher rate of recurrence in adolescents and young women than in premenopausal women. This higher recurrence rate for early endometriosis may be due to higher plasma estrogen levels or a more aggressive disease. Nevertheless, risk predictors for recurrence of disease cannot be detected [57]. According to the literature, postoperative recurrence of ovarian cysts and pain symptoms have been observed in 40–50% of cases at 5-year follow-up in young women not on hormonal therapy [58]. Therefore, all adolescents should undergo medical treatment after surgery, since hormonal therapy can prevent endometrioma recurrence, reducing the need for repeated surgery which increases damage to healthy tissue and reduces ovarian reserve [10]. Due to the poor efficacy of medical treatments, patients with severe painful symptoms and with deep infiltrating endometriosis can undergo surgery to restore the anatomy and improve the quality of life [59,60]. The surgery itself often causes important side effects [61]. Due to surgical procedures, fibrotic tissues, adhesions, and residual disease can be expected after DIE resection [62,63]. Therefore, in adolescent patients with chronic pelvic pain, offering surgery to confirm endometriosis disease diagnosis is considered inappropriate [64]. With respect to recent studies [17,65], in adolescents and young women, the endometriotic foci are small, and often isolated disease findings. The most frequent features are small USL thickening, mild adenomyosis, and small endometrioma of less than 30 mm, and are associated with severe dysmenorrhea. Thus, surgical management should be performed only when it is clinically required, such as in patients not responsive to medical therapy. Furthermore, adolescents should be made aware about the potential harmful effect of endometrioma, and at the same time, the possible impact of surgery on ovarian reserve and future fertility.

## 9. Follow-Up

According to the revised American Society of Reproductive Medicine (rASRM) staging classification, the severity of endometriosis is not related to the symptoms, treatment response, or prognosis [66]. Although the natural history of endometriosis is unknown, different types of lesions can vary across the life course [67].

There is no strong evidence that supports an ordered progression of endometriotic lesions. Since classification and staging systems have failed to provide algorithms for progression risk or prognosis, there are no guidelines recommending follow-up times to reduce the progression of the disease, either for patients on medical therapy or not. Despite these limitations, it could be useful to carry out checks in these patients to evaluate the benefit of medical or surgical therapy. From this point of view, it could be useful to re-evaluate these young girls every 4–6 months in the first year, and then once a year, in the absence of changes in pain symptoms.

Through this follow up, the clinician could modify the therapy and adapt it to the individual patient in order to apply tailored therapies for every patient.

## 10. Conclusions

Endometriosis in adolescents and young women represents a problem with many variables requiring consideration which differ from adult women. Underestimating dysmenorrhea, especially if severe, and the absence of indicative symptoms of endometriosis can delay the diagnosis. These delays could lead to disease progression and impact on the fertility of these young girls. Presumably, early diagnosis and appropriate management could lead to less major surgery in adulthood. Dysmenorrhea, and particularly severe dysmenorrhea, should be considered as important in schools and other educational institutions. In adolescents and young patients, small isolated disease findings are often common features of endometriosis. Therefore, adolescents and young women with dysmenorrhea should be referred to dedicated endometriotic centers for accurate noninvasive diagnosis and adequate medical and/or surgical management, as well as appropriate follow-up.

## Data Availability

The data are available from the corresponding author upon reasonable request.
